# Sustaining Sporting Destinations through Improving Tourists’ Mental and Physical Health in the Tourism Environment: The Case of Korea

**DOI:** 10.3390/ijerph17010122

**Published:** 2019-12-23

**Authors:** Yunduk Jeong, Suk-Kyu Kim, Jae-Gu Yu

**Affiliations:** 1 Department of Sport Management, Kyonggi University, Suwon 16277, Korea; fcgangwon@nate.com; 2 Department of Sports Science, Dongguk University, Gyeongju 38066, Korea; skkim2018@dongguk.ac.kr; 3 Department of Sport Industry, Chungang University, Anseong 17546, Korea

**Keywords:** emotional experiences, novelty seeking, tourist satisfaction, destination loyalty, active sport tourism

## Abstract

The purpose of this study was to explore structural relationships between emotional experiences, novelty seeking, tourist satisfaction, and destination loyalty in the context of active sport tourism. The study emphasizes the mediating effect tourist satisfaction has on the relationship between emotional experiences and destination loyalty. The validities and reliabilities of the measures used were examined through confirmatory factor analysis (CFA) and correlation analysis using 230 domestic and international participants who attended a marathon race as amateur athletes. Structural equation modeling analysis with maximum likelihood estimation was conducted to investigate relationships between study variables. Findings disclosed the positive impacts of (a) emotional experiences on tourist satisfaction and destination loyalty, (b) novelty seeking on tourist satisfaction, and (c) tourist satisfaction on destination loyalty, and demonstrated that (d) tourist satisfaction fully mediates the relationship between emotional experiences and destination loyalty. Based on its results, this study (a) indicates that emotional experiences play key roles in predicting tourist satisfaction and destination loyalty, (b) provides an example of the merits of the Destination Emotion Scale (DES) in a sport tourism setting, (c) implies that both emotional experiences and novelty seeking should be incorporated into tourist behavior models, and (d) contributes to tourism studies by exploring the mediating effect of tourist satisfaction on the relation between emotional experiences and destination loyalty. Thus, destination managers should manage gorgeous natural views and beautiful cityscapes, and organize various fun events, such as prize and ticket giveaway events, music performances, and charity campaigns for tourists during events.

## 1. Introduction

Stiff competition between destination management organizations with respect to attracting new and retaining loyal tourists in a saturated global marketplace has caused these organizations to devote time and resources to building tourist satisfaction and destination loyalty [1]. Tourist satisfaction is becoming of prime concern to destination marketers because it has long been recognized as an important component of trip experience and the possible cornerstone of future success [2]. For decades, destination managers have viewed destination loyalty as a critical element because it is considered to be more cost-effective than traditional marketing strategies [3]. In the past, marketing managers have focused on soliciting new customers through much time and effort. However, recent emphasis has been directed at the retention of loyal customers because it offers a less costly approach [4]. If tourists show great loyalty to a destination, they are more likely to easily disseminate throughout the Internet favorable opinions and experiences from visiting the destination, particularly through social media, such as personal blogs, Facebook and Instagram accounts, and via YouTube. That enables potential tourists to gain knowledge about a destination, and consequently, destination managers can reduce marketing budget expenses. Thus, building destination loyalty is now considered a cost-effective means of attracting tourists, and studying which factors drive destination loyalty is required in order to develop or sustain tourist destinations [3].

In the context of broader tourism literature, and in order to explore antecedents of tourist satisfaction and destination loyalty, the developments of destination image [5] and motivation [6] are commonplace aspects of research agendas. However, tourism researchers have recently turned their attentions to the testing of “global” and “integrative” models to improve theory development and application [7]. Following this recent academic trend, some scholars have suggested that understanding tourists’ emotional experiences is an important prerequisite for developing successful destinations [8]. This point of view has renewed the importance of considering emotion in the marketing literature over the past few years in agreement with the notion that “mental states of readiness arise from appraisals of events or one’s own thoughts” [9]. According to a recent thorough study [8], emotions are associated with tourist motivation and destination choice at the pre-trip stage and play a pivotal role in forming memorable experiences during trips and, ultimately, may influence tourist satisfaction and destination loyalty at the post-trip stage. This suggests that destination marketers should focus on tourism resource developments that evoke tourists’ emotions.

Sport tourism has received much attention from scholars for many years, because it has been generally recognized to provide an effective means of stably enhancing the growth of tourist destinations [10,11]. Gibson [10], who provided an exhaustive analysis of sport tourism, views sport tourism as “leisure-based travel that takes individuals temporarily outside of their home environments to participate in physical activities, to watch physical activities, or venerate attractions associated with physical activities”. Over recent years, several researchers have become increasingly interested in studying active sport tourism as a means of attracting and satisfying tourists [12]. Active sport tourism signifies that individuals travel to sporting events (such as windsurfing, canoe, golf, hiking, and skiing competitions) or participate in them (e.g., marathon, cycling, or triathlon sporting events) [13]. Moreover, according to Kaplanidou and Vogt’s study [14], active sport tourism provides tourists with emotional experiences such as excitement, relaxation, enjoyment, self-fulfillment, and pride, and that participation in a sporting event is an accomplishment and “a lot of fun”. Hence, it is clearly important that both academia and industry managers understand the role played by active sport tourism in the formation of tourists’ emotions.

Although foundations for sport tourism research have been constructed by the majority of previous studies, these foundations are limited in three important ways. First, the influence of novelty seeking on tourist satisfaction and destination loyalty remains a significant gap in sport tourism research. Novelty seeking is a key component of tourist motivation and in opposition to familiarity [15]. A considerable number of tourism researchers have concentrated on this concept over recent decades because it plays a central role in tourist decision-making and could lead to tourist satisfaction and destination loyalty [15,16]. Surprisingly, despite the need to understand novelty seeking, scant empirical work in the sport tourism literature has addressed direct relationships between novelty seeking and its consequences. Wong and Tang [17] suggested that since novelty seeking offers tourists thrills and surprises, destination managers and event organizers should utilize the notion to form loyalty.

Second, although studies have been conducted to understand emotional experiences and novelty seeking in the broader tourism context, few have sought to identify the mediating effects of tourist satisfaction on relations between emotional experiences and destination loyalty or between novelty seeking and destination loyalty. Rather, a considerable number of studies have focused on direct links between emotional experiences, novelty seeking, tourist satisfaction, and destination loyalty and have ignored the mediating role of tourist satisfaction on relations between such variables [16,17,18]. Drawing on the results of previous studies regarding the direct effects of these four variables, tourists would seem to be likely to regard “tourist satisfaction” as a significant component of the paths between emotional experience and destination loyalty, and between novelty seeking and destination loyalty.

Third, in a sport tourism context, a number of studies have concentrated on large-scale sporting events such as the Summer or Winter Olympic Games, the Fédération Internationale de Football Association (FIFA) World Cup, and World Championships [19]. However, few recent studies have focused on small-scale recurring sporting events. According to Wong and Tang [17], small-scale sporting events could also attract tourists and media coverage. Likewise, Jeong and Kim [20], who identified a suitable model of destination image in the sport tourism literature, mentioned that hosting small-scale recurring events might provide an important strategy for increasing the likely success of a sporting destination.

Accordingly, the main objective of this study was to explore mutual relationships between emotional experiences, novelty seeking, tourist satisfaction, and destination loyalty collectively and systematically with a view toward enhancing the competitive advantage of sporting destinations.

## 2. Theoretical Background, Research Hypotheses, and Model

### 2.1. Information about Cheonan

**Figure 1:**
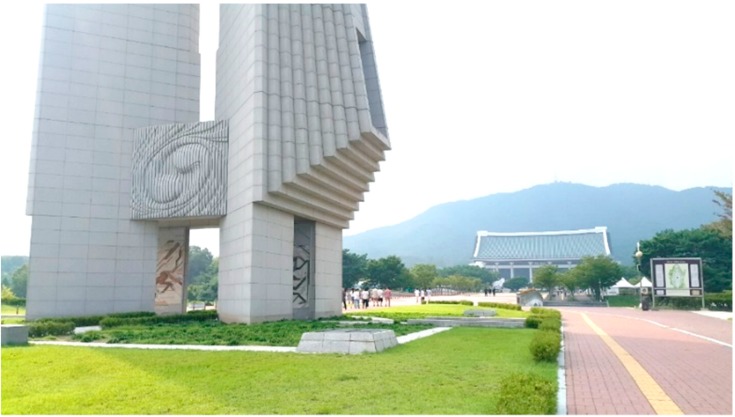
The Independence Hall in South Korea. Source: author’s compilation.

Cheonan is located in the northeastern area of Chungcheongnam-do Province in South Korea and in 2019 had a population of 678,764 [21]. With abundant cultural infrastructure, the city provides tourists with many opportunities for sightseeing. “The independence Hall of Korea” is the most popular site for tourists as it displays a range of Korean historical documents and relics with emphasis on the independence movements of the Japanese colonial period [22] (Figure 1). Cheonan is also well-known for its “Heungtaryeong Festival”, a dance festival centered on a unique style of traditional singing in Cheonan called Heungtaryeong, which features traditional dances from all corners of the world [23]. In addition, tourists love “Hodu-gwaja”, commonly translated as walnut cookies, walnut cakes, and walnut pastries, which are produced in the area, and “Sundae-gukbap”, a Korean soup made from sundae, rice, onions, sesame leaves, cabbage, and other vegetables [24].

Cheonan has focused on becoming a sporting destination in recent years. For example, the city is home to the “Cheonan Hyundai Capital Skywalkers Volleyball Club” and has hosted many sporting events to attract tourists. Recently, with growing awareness of health, increasing focus has been placed on marathon races, that is, on active sport tourism. “The Cheonan Ryu Gwansun Peace Marathon” is named after an artist and well-known independence activist, who supported efforts to rid Korea of Japanese oppression during the colonial period [25]. This marathon is an annual event and attracts around 10,000 spectators and participants [26].

### 2.2. Emotional Experiences

Emotions have been discussed by psychology and marketing research scholars for a considerable time because the concept is seen as the most critical driver of consumer behavior [27]. However, the interchangeable use of the terms affects, emotion, and mood constitutes a major problem. According to Hosany and Gilbert [28], these terms can be differentiated. The literature indicates that researchers view affect as “an umbrella term, and moods and emotions as instances of this feeling state”, and that moods are “mild affective states that are easily induced and not attributable to specific stimuli or objects, but rather transient, pervasive feeling states”. On the other hand, emotions are depicted as “affective states characterized by episodes of intense feelings that are linked with a specific referent such as a person, an object, or an event” [29]. For instance, active tourists can experience joy when achieving their personal goals, or at a tourism destination in response to music and dance. Thus, in this study, we adopt conceptual distinctions between emotion, affect, and mood.

Two major theoretical approaches to emotional experiences are evident in psychology literature, that is, dimensional (valence-based) and categorical (emotion specificity) approaches. Dimensional approaches include a handful of dimensions such as positive, negative, pleasure, and arousal, whereas categorical approaches view emotions as a set of idiosyncratic affective states (e.g., fun, pleasure, anger, sadness, and surprise) [8]. In the context of tourism literature, many researchers have focused on the valence-based approach to measure emotions and have used summary dimensions. For example, Grappi and Montanari [30] tested relationships between emotions, hedonic value, social identification, satisfaction, and repatronizing intention among visitors at an Italian festival and, taking valence into account, identified two dimensions of visitors’ emotions—positive and negative. Similarly, Bigné et al. [31] analyzed how visitor emotions in a theme park environment lead to satisfaction and behavioral intentions and concluded emotions consisted of two dimensions, namely pleasure and arousal, which are commonly used to explain emotions.

Moreover, Hosany and Gilbert [28] pointed out the applicabilities of existing psychologic emotion scales such as Mehrabian and Russell’s Pleasure, Arousal and Dominance Scale in 1974, Izard’s Differential Emotions Scale in 1977, Plutchik’s eight primary emotions in 1980, and Watson, Clark, and Tellegen’s Positive Affect and Negative Affect Scale in 1988, which remain suspect in terms of their applicabilities, reliabilities, and validities when applied to consumer studies [28]. In other words, emotion scales from psychology inadequately reflect the complexities of positive emotions [28,32]. As a result, the authors [28] developed the “Destination Emotion Scale” (DES), which represents the three emotional dimensions of joy, love, and positive surprise. Based on previous studies, Prayag et al. [8] viewed joy as an emotion evoked by positive outcomes such as success and good fortune, which may be key contributors to tourist experience. The feeling of love is a recognized important factor in the context of establishing that consumers are passionate about a brand or product [33]. Finally, the last dimension, surprise, contains emotion items such as amazement, astonishment, and inspiration, which arise from unexpected events [34].

In the active sport tourism setting, tourists can feel joy when they meet personal goals, improve mental and physical health, build self-confidence, relieve stress, learn new skills, and have unique experiences [35]. For instance, marathon participants run for distances of 5, 10, or 21 km, which enable them to enjoy natural views and beautiful cityscapes that elicit a feeling of love toward a city. Similarly, tourists that participate in surfing, scuba diving, golf, cycling, rock-climbing, or skiing develop love of the cities involved. Finally, positive surprise may be evoked from unexpected hospitality at events. For example, this occurs when a tourist experiences unexpected kindness from event volunteers, hotel or restaurant staff, taxi or bus drivers, or local residents. In addition, impromptu events such as dance or music performances, fan signings, or giveaway events can also elicit joy. However, since small-scale sporting events like marathon races involve short-term relationships with destinations, tourists generally have fewer opportunities to feel positive surprise. Hence, in the present study, positive surprise was excluded from the dimensions of emotional experience.

### 2.3. Novelty Seeking

Some tourists may desire a low level of novelty on a holiday, but the majority prefer a high level as they typically seek novel or unique experiences [36,37]. For this reason, novelty seeking has attracted much attention in tourism literature and many researchers have explored the definition of novelty seeking. Novelty seeking, a key component of travel motivation, is regarded as unfamiliar [37,38]. One of the most cited definitions is that “novelty seeking is the outcome of contrast between current perception and past experience” [39]. According to Lee and Crompton [37], novelty includes the constructs of adventure, boredom alleviation, surprise, and thrill, which indicates that novelty seeking is linked with variety seeking [36]. In the sport tourism setting, novelty seeking is considered a cornerstone of marketing and tourist retention. Wong and Tang [17] investigated the effects of travel motives and purposes on loyalty in the context of a smaller-scale sporting event, and noted novelty seeking can offer sport tourists an opportunity to change their daily routines, and that this leads to event loyalty.

### 2.4. Research Hypotheses

Previous studies have consistently reported emotions are critical for the building of satisfaction and loyalty [40]. Lee et al. [41] examined relationships between festivalscapes, patron emotions, satisfaction, and loyalty in a theoretical model, and proposed that positive emotions are direct antecedents of satisfaction and loyalty and that festival managers should strive to create positive experiences to evoke positive emotions. Faullant et al. [42] explored a conceptual model of personality, basic emotions, and satisfaction in the context of a mountaineering tour, and found that joy importantly boosts satisfaction. In addition, in a study on the influence of restaurant stimuli on diners’ emotions and loyalty, Peng et al. [43] concluded that positive emotions critically influence loyalty. Therefore, we propose the following hypotheses concerning the impacts of emotional experiences on tourist satisfaction and destination loyalty.

**Hypothesis** **1** **(H1).** Emotional experiences positively influence tourist satisfaction.

**Hypothesis** **2** **(H2).** Emotional experiences positively influence destination loyalty.

Supportive evidence exists for positive relations between novelty seeking, satisfaction, and loyalty. Toyama and Yamada [44] investigated differences between the effects of novelty and familiarity on satisfaction and destination loyalty, and emphasized that novelty plays a key role in predicting satisfaction and destination loyalty. The authors suggested that destination marketers should offer something new to target markets. Assaker et al. [16] analyzed the effect of novelty seeking, satisfaction, and destination image on tourist revisit patterns, and demonstrated that novelty seeking strengthens satisfaction. Given the positive impacts reported in previous studies, we considered that novelty seeking could positively influence tourist satisfaction and destination loyalty.

**Hypothesis** **3** **(H3).** Novelty seeking positively influences tourist satisfaction.

**Hypothesis** **4** **(H4).** Novelty seeking positively influences destination loyalty.

A vast amount of literature is devoted to the positive relationship between tourist satisfaction and destination loyalty. Eid [45] investigated relationships between perceived value, satisfaction, loyalty, and retention in the tourism industry, and confirmed that satisfaction is a primary antecedent of loyalty. In a study on structural relationships between destination image, perceived value, tourist satisfaction, and loyalty, Ramseook-Munhurrun et al. [46] presented evidence that tourist satisfaction leads to loyalty. In the context of active sport tourism, Jeong et al. [11] tested a theoretical model containing event quality, tourist satisfaction, place attachment, and behavioral intentions, and showed that tourist satisfaction is more inclined to build behavioral intentions. Based on empirical perspectives described in the literature, we postulated the following:

**Hypothesis** **5** **(H5).** Tourist satisfaction positively influences destination loyalty.

When exploring relations between emotions and loyalty or behavioral intentions, previous studies have considered satisfaction a key construct [47]. Martin et al. [47] examined the role of emotions in determining customer satisfaction and future behavioral intention in the context of service marketing and found that emotions influence satisfaction and, eventually, behavioral intention. Likewise, Walsh et al. [48] provided useful additional evidence on the intervening role of satisfaction on relations between emotions and loyalty, and reported that satisfaction partially mediates relations between emotions and behavioral intentions. Thus, it can be inferred that tourist satisfaction mediates the relation between emotional experiences and destination loyalty. With respect to the mediating effect of tourist satisfaction on the relationship between novelty seeking and destination loyalty, Albaity and Melhem [36] demonstrated that novelty seeking has a direct impact on destination loyalty by mediating satisfaction with a destination. Therefore, based on exhaustive former studies, we proposed the following hypotheses.

**Hypothesis** **6** **(H6).** Tourist satisfaction mediates the relations between emotional experiences and destination loyalty.

**Hypothesis** **7** **(H7).** Tourist satisfaction mediates the relationship between novelty seeking and destination loyalty.

**Figure 2:**
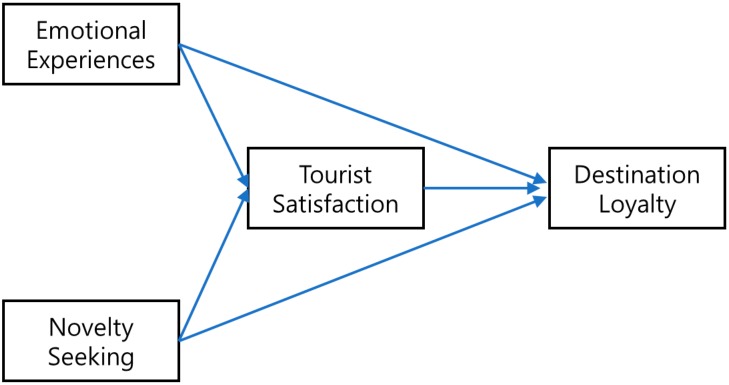
The proposed conceptual model. Source: author’s compilation.

Based on past studies, the present study offers the following conceptual model (Figure 2).

## 3. Materials and Methods

### 3.1. Data Collection

Data for the present study were collected from domestic and international participants that attended the 2019 Cheonan Ryu Gwansun Peace Marathon in Cheonan. Cheonan was chosen as the site for this study primarily for two reasons. First, the event is reportedly one of the most famous small-scale sporting events in South Korea. Second, Cheonan has a high level of repeat visitation among tourists. Thanks to its rich cultural heritage, the city has become one of the most famous tourist destinations in Korea. To obtain a sample, the authors and two trained research assistants administered a face-to-face questionnaire-based survey near the ticket office on 19 May 2019, using a convenient sampling procedure. We approached 270 respondents and asked them to participate in the survey without reward; participants were not actively solicited to participate and locals were totally excluded in the survey. Consequently, 250 questionnaires were collected, but 20 were subsequently eliminated because some important questions were not answered. The remaining 230 satisfactorily completed questionnaires were analyzed. The sample contained 165 (71.7%) males and 65 (28.3%) females, and 140 (60.9%) domestic tourists and 90 (39.1%) international tourists. Of the respondents, 78 (33.9%) were aged between 40 and 49 years, 81 (35.2%) were university educated, 144 (62.6%) were married, and 47 (20.4%) earned between $40,000 and $59,999 per annum.

### 3.2. Measures

The survey questionnaire consisted of five main sections: (a) emotional experiences, (b) novelty seeking, (c) tourist satisfaction, (d) destination loyalty, and (e) respondent demographic information. Emotional experiences’ items were adapted from Hosany and Gilbert’s Destination Emotion Scale (DES) [28]. The DES consists of three dimensions (joy, love, and positive surprise) representing tourists’ emotional experiences. Joy was measured using three items (delight, joy, and pleasure) and love was also captured with three items (caring, love, and affection). As discussed before, positive surprise was excluded from the dimensions of emotional experience because of short-term relationships between tourists and a small-scale sporting event. Several studies have confirmed the validity and reliability of DES in tourism [8]. Novelty seeking was assessed using four items derived from those used by Albaity and Melhem [36], and Chang et al. [49]: “This destination offers an unusual experience”; “This destination offers new discoveries”; “This destination offers new experiences”; and “This destination is new for me”. Albaity and Melhem [36] evaluated the validity and reliability of the items in tourism and indicated that all factor loadings were greater than 0.55 and Cronbach’s alpha coefficient was more than 0.7. Tourist satisfaction and destination loyalty were each assessed using three items adapted from Albaity and Melhem [36]: “I am really satisfied with the decision to visit Cheonan”; “I prefer this destination to others”; “This experience is exactly what I needed”; “I intend to visit Cheonan in the future”; “Cheonan provides more benefits than other destinations”; and “I would recommend other people to visit Cheonan”. The researchers calculated all factor loadings and Cronbach’s alpha coefficient, indicating that all the items and constructs were satisfactory. Items were scored using a five-point Likert scale ranging from 1 to 5 (strongly disagree to strongly agree).

Once a preliminary questionnaire to measure each construct was established, a panel of six experts (sport tourism and management professors) reviewed the scales for content validity. Items were evaluated based on whether the format and constructs were (a) appropriate, (b) adequate/representative, and (c) accurate/clear. Based on the feedback provided by the panel of experts, the preliminary questionnaire was modified, revised, and improved mainly in the areas of adequacy, test format, factor relevance, and wording clarity.

### 3.3. Validity and Reliability

A confirmatory factor analysis (CFA) model with a total of 98 degrees of freedom was used to assess the goodness of fit of the measurement model by using the latest version of AMOS software (22.0, IBM, New York, NY, United States). The CFA model showed that the measurement model reasonably fitted data (x^2^/*df* = 2.600, IFI = 0.931, TLI = 0.914, and CFI = 0.930). All model fit indices were within recommended thresholds [50]. Construct validity was assessed using convergent and discriminant validities. For convergent validity, we calculated factor loadings, construct reliability (CR), and average variance extracted (AVE). All factor loading values were >0.55 and significant (*p* < 0.001) [51] (Table 1). CR values all exceeded the recommended value of 0.7 (range from 0.928 to 0.951) and AVE values all exceeded the minimum requirement of 0.5 (range from 0.687 to 0.922) [52]. Hence, the convergent validity of emotional experiences, novelty seeking, tourist satisfaction, and destination loyalty was satisfactory (Table 1).

For discriminant validity, we verified that the AVE of the latent variable was greater than the square of the correlation coefficient between latent variables. Since it was difficult to verify all variables, the pair with the highest correlation coefficient was selected and verified. The highest correlation coefficient was 0.466 (emotional experiences–tourist satisfaction) (Table 2) and the square of 0.466 was 0.217. The AVE of emotional experiences was 0.687 and that of tourist satisfaction was 0.816 (Table 1). Since AVE values were all greater than the square of the highest correlation coefficient (0.687 and 0.816 > 0.217), discriminant validity was satisfactory.

Reliability estimates (Cronbach’s alpha) for emotional experiences, novelty seeking, tourist satisfaction, and destination loyalty were all above the recommended threshold of 0.7 (range from 0.849 to 0.922), which indicated measures were reliable [52] (Table 1).

## 4. Results

The five hypothesized relationships were analyzed by structural equation modeling (SEM). Overall, the SEM achieved acceptable fit (x^2^/*df* = 1.797, *p* < 0.001). Absolute fit index (Root Mean-square Residua = 0.030, Goodness of Fit Index = 0.921, and Root Mean Square Error of Approximation = 0.059) and incremental fit index (Normed Fit Index = 0.927, Relative Fit Index = 0.909, Incremental Fit Index = 0.966, Tucker–Lewis Index = 0.957, and Comparative Fit Index = 0.966) were satisfactory [51].

Estimates of structural coefficients (paths) provided the basis for testing the proposed hypotheses. As shown in Table 3, emotional experiences had a significantly positive effect on tourist satisfaction (0.415, *p* < 0.001) and destination loyalty (0.183, *p* < 0.05), which offers supportive evidence for hypotheses 1 and 2. The path coefficient from novelty seeking to tourist satisfaction was positive and statistically significant (0.190, *p* < 0.05), supporting hypothesis 3. Non-significant paths emerged for novelty seeking → destination loyalty, which rejected hypothesis 4. However, hypothesis 5 was supported, as tourist satisfaction significantly and positively influenced destination loyalty (0.302, *p* < 0.001).

To identify effects mediating tourist satisfaction, we utilized the bootstrap test, which is a data-based resampling statistical method [53]. As regards the mediating effect of tourist satisfaction on the relation between emotional experiences and destination loyalty, the direct effect included a zero point and was not significant, but the indirect effect did not include a zero point and was significant (Table 4). Thus, tourist satisfaction was found to have a full mediating effect, which supported hypothesis 6. Regarding the mediating effect of tourist satisfaction on the relationship between novelty seeking and destination loyalty, the direct effect included a zero point and was not significant, and the indirect effect was also not significant. Hence, tourist satisfaction did not show any mediating effect, which contradicted hypothesis 7. In summary, only the relationship between emotional experiences and destination loyalty via tourist satisfaction indicated mediation.

## 5. Discussion

From a theoretical point of view, the current study offers several contributions to research in marketing, tourism, and sport marketing. First, this study answers the recent call of Prayag et al. [8] to explore the roles played by emotional experiences on tourist satisfaction and destination loyalty. Those authors expanded current theorizations by investigating the merits of emotions in tourist behavior models, emphasized that emotions are central to the understanding of satisfaction and behavioral intentions, and suggested that these relations be investigated in other tourism settings. Our findings establish that emotional experiences have a positive effect on tourist satisfaction and destination loyalty in the context of active sport tourism, as initially identified in Prayag et al. [8]. In other words, this study provides strong support for the notion that emotional experiences directly affect tourist satisfaction and destination loyalty, which is in line with previous studies [41,42,43]. Thus, this study strengthens the view that sport tourists’ emotions should be considered a priority by sporting destination marketers.

Second, from a methodological perspective, our results provide an example of the merits of the Destination Emotion Scale (DES) developed by Hosany and Gilbert [28] in a sport tourism setting. According to Prayag et al. [8], a valence-based psychologic approach (e.g., positive or negative, or pleasure or arousal) has been favored in previous tourism studies. However, such traditional approaches neglect the complexity of tourists’ emotions, fail to secure consequences of some valence emotions (e.g., joy and love), and finally raise serious concerns regarding validity in the tourism literature [8,28]. For these reasons, Hosany and Gilbert [28] created a DES representing three emotional dimensions (joy, love, and positive surprise). Our findings indicate that convergent validity, discriminant validity, and reliability of DES are satisfactory and reliable in the context of sport tourism. Thus, it is advised that sport tourism researchers planning to conduct research on tourists’ emotions consider adopting DES as an emotion scale.

Third, the current study responds to a recent call to use integrative models [54]. Accordingly, we incorporated both emotional experiences and novelty seeking in a tourist behavior model, and indicated novelty seeking has a favorable influence on tourist satisfaction. Previous studies support a link between novelty seeking and satisfaction [36,44,45]. For instance, Lee et al. [55] developed a theoretical model for relationships between service encounters, physical environment performance, novelty, overall satisfaction, and loyalty in a cruise tourism context, and confirmed that novelty seeking critically contributes to the building of satisfaction. This finding indicates that if sport tourists consider a destination novel, they are more likely to feel satisfied. For this reason, Albaity and Melhem [36] argued it is important that destination managers develop special destinations that provide tourists with truly unique and novel experiences.

Fourth, this study contributes to tourism studies by identifying the mediating effect of tourist satisfaction on the relation between emotional experiences and destination loyalty. Prior studies failed to address the mediating effect of tourist satisfaction; for example, Kim and Lennon [56] investigated the relationships between reputation, website quality, emotion, perceived risk, and purchase intention and showed that emotion has a positive effect on purchase intention. However, they overlooked the mediatory role of satisfaction on the relationship between emotion and intention. Our results highlight the need to incorporate satisfaction and emotion in models aimed at determining tourist loyalty or intentions. Notably, our findings reveal that tourist satisfaction has a full mediating effect on the relationship between emotional experiences and destination loyalty. This result is important because level of satisfaction necessarily intervenes in the relationship emotion and loyalty. By exploring the mediating effect of tourist satisfaction, the current study helps to address a gap in the marketing and tourism literature.

Fifth, this study stresses the importance of hosting small-scale sporting events for developing or sustaining sporting destinations. While a number of studies have been dedicated to large-scale sporting events, only a handful of studies highlight the merits of small-scale sporting events. According to Kim [57], despite the positive aspects of large-scale sporting events, such events are still recording large financial deficits; therefore, host communities are forced to shoulder a heavy financial burden afterwards. On the other hand, small-scale and recurring sporting events could attract tourists and media coverage without an enormous cost [20]. The present study indicates that emotional experiences through small-scale sporting events have significant positive effects on tourist satisfaction and destination loyalty. Indeed, it is likely that small-scale and recurring sporting events in which active sport tourists participate as amateur athletes could provide tourists with unique, extraordinary, and memorable experiences, such as improving mental and physical health, building self-confidence, relieving stress, meeting personal goals, and competing with others [20]. Therefore, tourism destination managers should not underestimate the marketing power of small-scale and recurring sporting events.

Contrary to our prediction, novelty seeking was not found to affect destination loyalty, and as such, the mediating effect of tourist satisfaction on the relation between novelty seeking and destination loyalty was not supported. One explanation for this discrepancy is that whereas non-sport tourists generally have long-term relationships with destinations, sport tourists that attend small-scale sporting events as participants have short-term relationships [20], and fewer opportunities to experience novelty seeking offered by destinations. Another reason for this discrepancy is that tourists tend not to return even if they are satisfied with a destination [58], but rather, tend to seek memorable or extraordinary experiences at new destinations [59]. However, as discussed, tourists’ emotional experiences drive destination loyalty, and thus, destination marketers should take note of the important role played by emotional experiences in the stimulation of tourist retention.

The major results of this study disclose that strengthening emotional experiences and novelty seeking cannot be isolated from strategies designed to generate tourist satisfaction or destination loyalty and provide practical hints to sport tourism managers or marketers. To generate love as a component of emotional experiences, active sport tourists should be exposed to gorgeous natural views and beautiful cityscapes. As we have discussed in the literature review section, marathon participants usually run a distance of 5, 10, or 21 km and have an hour or less to appreciate spectacular architecture, picturesque villages, or unique natural environments, which can elicit a feeling of love toward a city. The study by Kulczyk-Dynowska and Bal-Domańska [60] noted that, since national parks located in a municipality provide a tourism function, municipalities should take advantage of their natural value for the sake of stimulating the regional economy. To incorporate joy among emotional experiences, various fun events should be organized for tourists during events. For example, prize and ticket giveaway events, music performances, charity campaigns, and local produce exhibitions can evoke a feeling of joy toward a destination. Ultimately, unflagging efforts to arouse feelings of love and joy might be achieved by offering tourists new or unusual experiences.

## 6. Conclusions

The objectives of this study were to determine the nature of relationships between emotional experiences, novelty seeking, tourist satisfaction, and destination loyalty with an emphasis on the mediating effects of tourist satisfaction in the tourism environment using an integrated model. The proposed model allowed the identification of relations between (1) emotional experiences and tourist satisfaction, (2) emotional experiences and destination loyalty, (3) novelty seeking and tourist satisfaction, and (4) tourist satisfaction and destination loyalty, and showed that tourist satisfaction had a full mediating effect on the relationship between emotional experiences and destination loyalty. The current study offers several contributions to research in tourism and sport marketing. For instance, this study answers the recent call of Prayag et al. [8] to explore the roles played by emotional experiences on tourist satisfaction and destination loyalty, provides an example of the merits of the Destination Emotion Scale (DES) developed by Hosany and Gilbert [28] in a sport tourism setting, responds to a recent call for tourism researchers to use integrative models [54] incorporating both emotional experiences and novelty seeking in a tourist behavior model, and contributes to tourism studies by identifying the mediating effect of tourist satisfaction on the relation between emotional experiences and destination loyalty. Thus, destination managers should manage gorgeous natural views and beautiful cityscapes and organize various fun events such as prize and ticket giveaway events, music performances, and charity campaigns for tourists during events.

Although the current study broadens our knowledge of emotions and novelty seeking concepts in tourism, it is subject to several limitations. First, it would have been interesting to include tourist involvement in evaluations of emotions, satisfaction, and loyalty. Degree of involvement in a marathon event or any other active sport tourism event is likely to influence emotions and play a major role in driving satisfaction and loyalty. Second, we considered satisfaction to be a mediator of the relation between emotional experiences and destination loyalty, and between novelty seeking and destination loyalty. However, we suggest future studies explore whether other mediators such as destination image and perceived value affect relationships between the factors. Third, these findings are limited to one sporting event, and data were collected at a specific place and time of year. Consequently, our findings cannot be generalized to active sport tourists. Future research could study active sport tourists at different sporting events and locations to increase the generalizability of the results. Fourth, because marathon races in which active sport tourists participate provide only short-term relationships with destinations, we did not measure the positive surprise component of emotional experiences. We suggest this dimension be included in future studies on sport tourism events, such as the Olympics, World Cup, and World Championships, that provide long-term relationships with destinations. Fifth, in this study, the likely impact on tourists’ experiences of volunteers’ behavior was not taken into consideration. Generally, sporting events have some level of contact between tourists/visitors and volunteers. Consequently, this situation offers opportunities for interaction, and thus might considerably influence tourists’ emotional experiences. Future research could examine the role of volunteers’ performance in predicting tourists’ emotional experiences.

## Figures and Tables

**Table 1:** Confirmatory factor analysis (CFA) results for the measurement model.

Scale Items	Standardized Loadings	CR	AVE	α
**Emotional Experiences**				
I feel a sense of Delight toward Cheonan	0.808	0.928	0.687	0.871
I feel a sense of Joy toward Cheonan	0.809			
I feel a sense of Pleasure toward Cheonan	0.802			
I feel a sense of Caring toward Cheonan	0.566			
I feel a sense of Love toward Cheonan	0.708			
I feel a sense of Affection toward Cheonan	0.628			
**Novelty seeking**				
This destination offers an unusual experience	0.871	0.930	0.770	0.895
This destination offers new discoveries	0.811			
This destination offers new experiences	0.831			
This destination is new for me	0.791			
**Tourist satisfaction**				
I am really satisfied with the decision to visit Cheonan	0.807	0.930	0.816	0.849
I prefer this destination to others	0.848			
This experience is exactly what I needed	0.776			
**Destination loyalty**				
I intend to visit Cheonan in the future	0.847	0.951	0.866	0.922
Cheonan provides more benefits than other destinations	0.893			
I would recommend other people to visit Cheonan	0.939			
x^2^ = 2.600, *df* = 98, IFI = 0.931, TLI = 0.914, and CFI = 0.930				

Source: author’s compilation. CR (construct reliability), AVE (average variance extracted), IFI (Incremental Fit Index), TLI (Tucker–Lewis Index), CFI (Comparative Fit Index).

**Table 2:** Correlations between constructs.

Construct	1	2	3	4
Emotional experiences	1			
Novelty seeking	0.354 **	1		
Tourist satisfaction	0.466 **	0.330 **	1	
Destination loyalty	0.344 **	0.233 **	0.394 **	1

Source: author’s compilation. ** *p* < 0.01.

**Table 3:** Structural parameter estimates.

Hypothesis	Path	Standardized Coefficient	*T*-Value	Supported?
1	Emotional experiences → Tourist satisfaction	0.451	5.492 ***	Yes
2	Emotional experiences → Destination loyalty	0.183	2.104 *	Yes
3	Novelty seeking → Tourist satisfaction	0.190	2.469 *	Yes
4	Novelty seeking → Destination loyalty	0.104	1.361 *	No
5	Tourist satisfaction → Destination loyalty	0.302	3.498 ***	Yes

Source: author’s compilation. *** *p* < 0.001, * *p* < 0.05.

**Table 4:** Effects mediating tourist satisfaction.

Path		Standardized Coefficient	95% CI (Bias-Corrected)	*p*
Emotional experiences → Tourist satisfaction → Destination loyalty	Direct effect	0.183	−0.025 to 0.409	0.181
	Indirect effect	0.136	0.033 to 0.267	0.012
	Total effect	0.320	0.122 to 0.481	0.015
Novelty seeking → Tourist satisfaction → Destination loyalty	Direct effect	0.104	−0.056 to 0.316	0.281
	Indirect effect	0.057	0.006 to 0.192	0.054
	Total effect	0.162	−0.040 to 0.361	0.162

Source: author’s compilation.
